# Association between triglyceride glucose index and endometriosis in adults in the United States: A comprehensive study from the National Health and Nutrition Examination Survey (NHANES)

**DOI:** 10.1371/journal.pone.0313601

**Published:** 2024-11-13

**Authors:** Sasa Gao, Xiaoping Cui

**Affiliations:** 1 Traditional Chinese Medicine Department, Northwest Women’s and Children’s Hospital, Xi ’an, Shaanxi Province, China; 2 Higher Education Center, Shaanxi University of Chinese Medicine, Xianyang City, Shaanxi Province, China; University of Bremen: Universitat Bremen, GERMANY

## Abstract

**Background:**

The triglyceride glucose (TyG) index has been well recognized as a reliable marker of insulin resistance and substantially correlated with the pathogenesis and progression of hypertension and cardiovascular diseases. However, no study has investigated the association between the TyG index and endometriosis. Therefore, this study aimed to uncover an association between the TyG index and endometriosis.

**Methods:**

This cross-sectional investigation employed the extensive dataset derived from the National Health and Nutrition Examination Survey (NHANES) (1999–2006). To explore the potential connection between the TyG and endometriosis, a multivariate weighted logistic regression model was established. The nonlinear relationship between the TyG index and the risk of endometriosis was explored using restricted cubic spline models (RCS). Furthermore, subgroup analyses were conducted.

**Results:**

Ultimately, 2,508 individuals were included in this investigation. The findings unveiled a robust positive correlation between the TyG index and the susceptibility to endometriosis (OR [95% CI]: 1.52 [1.024,2.258]; P < 0.05). This positive association remained consistent across diverse subgroups. Age, birthplace, and whether one ovary was removed were identified as significant risk factors. In RCS analysis, the TyG index showed a nearly linear relationship with the risk of endometriosis (P-nonlinear > 0.05).

**Conclusions:**

The findings indicate a positive association between the TyG index and the risk of endometriosis, exhibiting an approximate non-linear relationship.

## 1.Introduction

Endometriosis represents a complex medical disorder characterized by a range of syndromes resulting from the abnormal implantation of endometrial stroma and glands outside the uterine cavity. Its most significant clinical manifestations are debilitating pain and infertility [1]. The pathogenesis of endometriosis remains a topic of considerable debate. The latest research evidence indicates that hormones and immune factors create an inflammatory environment that promotes the onset and development of endometriosis [2,3]. Additionally, the activation of inflammatory signal transduction pathways is closely related to insulin resistance (IR) [4].

Nevertheless, the association between IR and endometriosis remains elusive. Studies have established that obesity is a prominent etiological factor of IR [5]. Hyperlipolysis, characterized by increased lipolysis, is a pivotal source of pro-inflammatory cytokines [6]. Obesity and IR are associated with low-grade chronic systemic inflammation. Adipose tissue promotes levels of pro-inflammatory factors, such as tumor necrosis factor-alpha (TNF-α), interleukin-1 (IL-1), and interleukin 6 (IL-6). These pro-inflammatory cytokines stimulate major inflammatory NFκB and JNK pathways in cells [7]. Consequently, in the presence of IR, inflammatory factors are produced. The development of endometriosis is associated with abnormalities in autoimmune functions, especially the function of immune cells, such as neutrophils, macrophage differentiation, T cells, and B cells [8]. In endometriosis, macrophages and mast cells have been found to release chemokines such as TNF and IL-6 [9,10]. These immune cells can promote endometriotic cell proliferation and neovascularization in lesions [11,12]. If the phagocytosis of cells by macrophages is weakened, the growth inhibition of ectopic endometrium is lifted. In addition, mast cells are important immune cells involved in allergic reactions in the body. The number of mast cells in ectopic endometrial tissue is substantially higher than that in normal endometrial tissue. More important differentiation factors of mast cells are observed in the abdominal cavity of patients with endometriosis [13,14]. Furthermore, NK cells and T cells play an essential role in endometriosis. This suggests that the abnormal immune response mediated by immune cells caused by IR is a key factor in endometriosis. The continuous release of proinflammatory mediators constitutes a risk factor for endometriosis. Accordingly, we hypothesize that IR is crucial in the pathophysiological development of endometriosis.

The triglyceride-glucose (TyG) index, an established biomarker of IR, can predict the risk of diabetes, hypertension, and coronary heart disease across diverse ethnic populations. It has been extensively studied as an indicator of IR, with significant associations with the prognosis in patients with acute coronary syndrome, heart failure, and other circulatory diseases [15,16]. Moreover, research has revealed that elevated levels of the TyG index are significantly correlated with the risk of gestational diabetes mellitus and gestational hypertension [17]. Nonetheless, the relationship between the TyG index and endometriosis remains inadequately explored. Thus, this study was to elucidate the potential association between the TyG index and endometriosis, based on the comprehensive NHANES database.

## 2. Materials and methods

### 2.1 Study population

**Fig 1:**
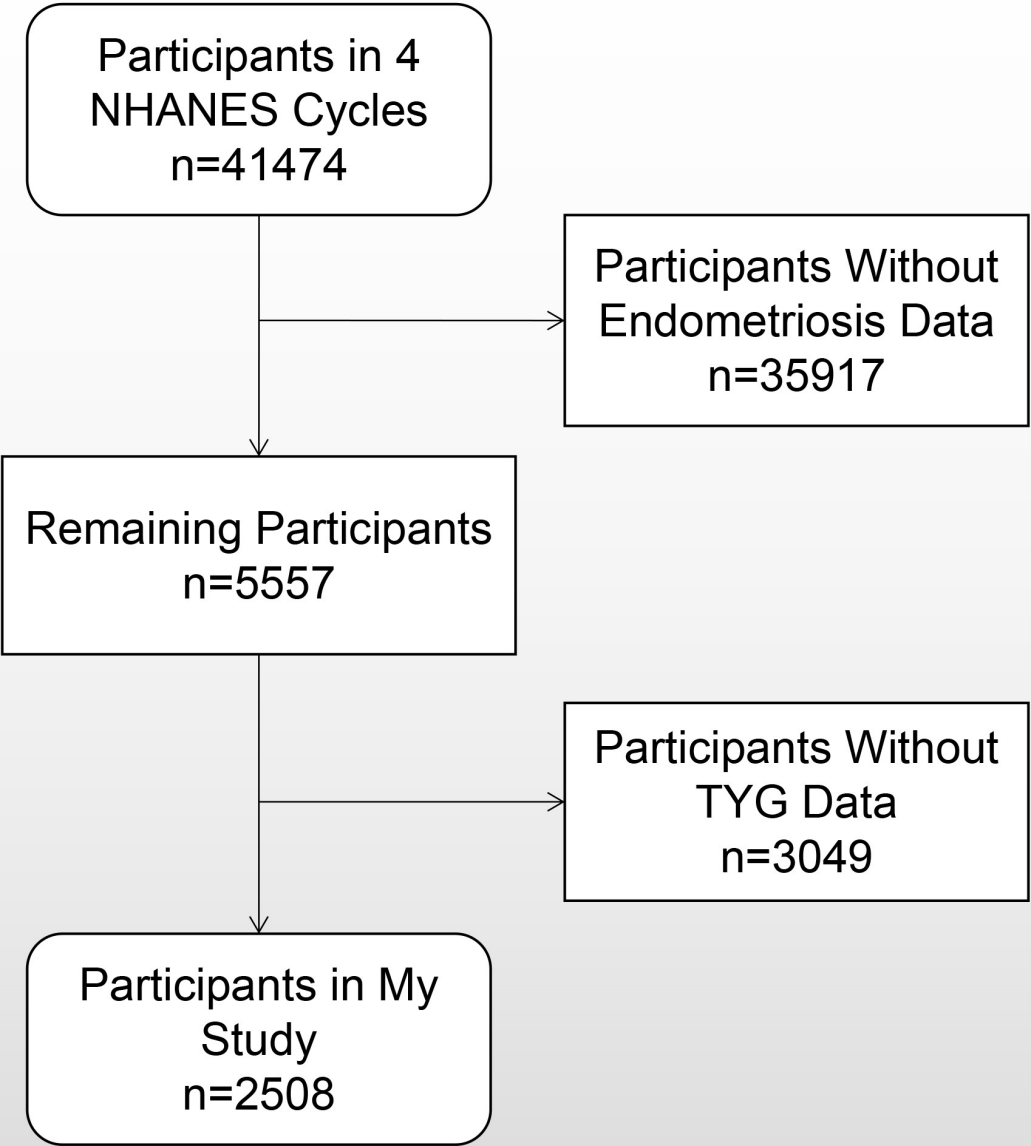
Flow chart of subject inclusion.

All data employed in this study are accessible through the NHANES website (https://www.cdc.gov/nchs/nhanes/). The NHANES is a population-based cross-sectional survey aimed at collecting detailed information on the health and nutritional status of adults and children in the United States. This survey integrates structured interviews and rigorous physical examinations. The dataset encompasses demographic particulars, dietary profiles, clinical examination findings, laboratory assays, questionnaire responses, and limited-access data. The findings from this survey are instrumental in assessing the prevalence of major diseases and associated risk factors, as well as in evaluating national parameters such as height, weight, and blood pressure. Only the study period of 1999–2006 had variables for endometriosis. This study analyzed data from the most recent four NHANES survey cycles from 1999 to 2006. Initially, the dataset comprised 41,474 subjects. However, in the process of data collection, 35,917 subjects were systematically excluded due to the absence of pertinent endometriosis data, 3,017 subjects due to lacking essential fasting glucose measurements, and an additional 32 subjects due to lacking fasting triglyceride data. Finally, 2,508 subjects were included. A detailed screening procedure is visually depicted in Fig 1.

### 2.2 Statement of ethics

Studies involving human participants were reviewed and approved by the NCHS Research Ethics Review Board (ERB). In accordance with national legislation and institutional requirements, written informed consent was not required for participation in this study.

### 2.3 Study variables

#### 2.3.1 Fasting triglyceride and glucose measurements

Triglyceride levels were assessed exclusively during the morning examination within the NHANES. This study included a cohort of individuals aged≥ 12 years who had fasted for at least 8.5 hours but less than 24 hours, yielding measurable triglyceride concentrations and non-zero fasting sample weights. Glucose concentration was measured utilizing the hexokinase method—an enzymatic assay for endpoint determination, involving the correction of sample blanks. Fasting blood glucose was tested in all participants in the morning after a fasting period of 9 hours.

#### 2.3.2 Definition of endometriosis

Data concerning endometriosis were sourced from the reproductive health questionnaire (RHQ). The RHQ is an intricate and comprehensive instrument that includes numerous questions, which are categorized by age or skip patterns based on responses to diverse reproductive health issues. Subjects were defined as having endometriosis if they received a diagnosis from a doctor or other professionals.

Specific information on other covariate data, such as age, education level, country of birth, BMI, weight, and marital status can be obtained at www.cdc.gov/nchs/nhanes/.

#### 2.3.3 Selection of the genetic instruments

The Mendelian randomization (MR) analysis for IR and endometriosis was obtained from the ieu database with the ids ebi-a-GCST005179 and ebi-a-GCST90018839, respectively. The ieu database provides 346,363,816,988 genetic associations from 50,040 GWAS summary datasets. MR was performed using the TwoSampleMR R software package with IR as the exposure and endometriosis as the outcome. Due to the small number of single nucleotide polymorphisms (SNPs) associated with IR or endometriosis, we selected instrumental variables with P values < 1x10^-5^, which were independent (R2 cutoff of 0.1, distance window, 10,000 KB).

### 2.4 Statistical analysis

Continuous variables were presented as the standard deviation (SD) of the mean or the mean of the interquartile range (IQR) and categorical variables were presented as frequencies and percentages (%). Statistical comparisons for continuous variables were conducted using Student’s t-test, whereas categorical variables were compared using Pearson’s chi-square test or Fisher’s exact test. According to the official NHANES guidelines, weights from 4-year fasting data were used from 1999 through 2002 (WTSAF4YR) and weights from 2-year fasting data were used from 2003 through 2006 (WTSAF2YR). TyG index was categorized into four quartiles and three models were established via weighted logistic regression. Model 1 remained unadjusted, Model 2 was partially adjusted (adjusted for age and BMI), and Model 3 was fully adjusted (adjusted for age, BMI, educational attainment, country of birth, gender, and age of the reference person, marital status of the reference person, age at menarche, parity, and whether at least one ovary was removed). To avoid multicollinearity, for the variables in the multifactor model, we calculated the variance inflation factor (VIF) for each variable in Model 3 and excluded variables with VIF > 5. A restricted cubic spline (RCS) model was employed to assess the non-linear correlation between the TyG index and the risk of endometriosis. Furthermore, subgroup analyses were performed by age and BMI, and forest plots were generated. All analyses were performed utilizing R 4.3.0 (https://www.r-project.org). P<0.05 denoted statistical significance.

The fixed‐effects inverse‐variance weighted (IVW) method was utilized to obtain the primary MR estimation. In the presence of horizontal pleiotropy in the SNPs, MR‐Egger regression and weighted median were conducted to test the robustness of the results. Isn the sensitivity analysis, IVW methods with Cochran’s Q statistics and MR‐Egger intercept were utilized to evaluate the heterogeneity and pleiotropy of individual SNPs. Statistical analyses were undertaken using the TwoSampleMR package in R statistical software 4.3.2. (R Foundation, Vienna, Austria), with a two‐tailed p‐value < .05 implying statistical significance.

## 3.Results

The characteristics of the included subjects are detailed in Table 1. 2508 subjects aged between 30 and 46 years were examined for fasting triglycerides and fasting glucose and answered questionnaires about endometriosis. Age, birthplace, age of the family reference person at the time of screening, fasting triglyceride, whether at least one ovary was removed, and TyG index were significantly different.

**Table 1:** The characteristics of participants.

Characteristic	Overall, N = 100,954,992^*1*^	0, N = 90,588,758^*1*^	1, N = 10,366,234^*1*^	p-value^*2*^
BMXBMI	27 (23, 32)	27 (23, 32)	27 (23, 32)	0.5
Female	2,508 (100%)	2,324 (100%)	184 (100%)	
RIDAGEYR	38 (30, 46)	37 (29, 46)	41 (35, 46)	<0.001
Country of Birth				<0.001
50 US States or Washington	1,931 (85%)	1,756 (83%)	175 (97%)	
Mexico	357 (4.9%)	357 (5.5%)	0 (0%)	
Elsewhere	220 (10%)	211 (11%)	9 (3.2%)	
Education Level-Adults 20+				0.06
Less Than 9th Grade	223 (4.6%)	221 (5.0%)	2 (0.9%)	
9-11th Grade (Includes 12th grade with no diploma)	384 (11%)	364 (11%)	20 (9.0%)	
High School Grad/GED or Equivalent	543 (23%)	497 (22%)	46 (29%)	
Some College or AA degree	820 (36%)	752 (35%)	68 (38%)	
College Graduate or above	538 (26%)	490 (26%)	48 (23%)	
Reference Person Gender				>0.9
Male	1,272 (51%)	1,178 (51%)	94 (51%)	
Female	1,236 (49%)	1,146 (49%)	90 (49%)	
Reference Person Age	41 (32, 49)	40 (32, 49)	43 (37, 49)	0.006
Reference Person Marital Status				0.12
Married	1,489 (61%)	1,370 (60%)	119 (67%)	
Widowed	78 (2.5%)	76 (2.7%)	2 (0.4%)	
Divorced	253 (11%)	229 (11%)	24 (15%)	
Separated	136 (4.2%)	125 (4.2%)	11 (4.5%)	
Never married	331 (13%)	312 (13%)	19 (7.4%)	
Living with partner	221 (8.5%)	212 (8.9%)	9 (5.5%)	
Doctor told you have diabetes				0.6
Yes	107 (4.0%)	99 (4.0%)	8 (3.6%)	
No	2,388 (96%)	2,213 (96%)	175 (96%)	
Borderline	13 (0.4%)	12 (0.5%)	1 (0.1%)	
Fasting Glucose	92 (86, 98)	92 (86, 98)	91 (86, 100)	0.8
triglyceride	99 (69, 146)	97 (69, 144)	114 (76, 178)	0.002
Age when first menstrual period occurred	13.00 (12.00, 14.00)	13.00 (12.00, 14.00)	12.00 (11.00, 13.00)	0.13
Number of pregnancies	2.84 (2.00, 3.58)	2.76 (2.00, 3.45)	3.00 (2.00, 4.00)	0.09
Had at least one ovary removed	197 (10%)	130 (6.9%)	67 (41%)	<0.001
TyG_num	8.42 (8.05, 8.85)	8.42 (8.04, 8.83)	8.56 (8.13, 9.06)	0.004

^**Notes:** *1*^ Median (IQR); n (unweighted) (%).

^*2*^ Wilcoxon rank-sum test for complex survey samples; chi-squared test with Rao & Scott’s second-order correction.

Three multivariate weighted logistic regression models were employed to examine the association between the TyG index and endometriosis (Table 2). A significant positive association was revealed between the TyG index and endometriosis in the unadjusted model (Model 1) and partially adjusted model (Model 2). After adjustment for full covariates (Model 3), this positive association remained robust (P = 0.038). The prevalence of endometriosis was notably increased in the highest TyG index group compared with the lowest TyG index group. Furthermore, among the covariates, age, birthplace, and whether an ovary had been removed had significant effects on the risk of endometriosis.

**Table 2:** Association between TyG index and endometriosis.

Characteristic	model1 OR (95%CI)	model2 OR (95%CI)	model3 OR (95%CI)
TyG (numeric)	1.676(1.293,2.172) ***	1.642(1.224,2.204) ***	1.52 (1.024,2.258) *
TyG(Quartile)			
Q1	Reference	Reference	Reference
Q2	1.599(0.942,2.717)	1.604(0.958,2.684)	1.749(1.032,2.964) *
Q3	1.553(0.831,2.903)	1.506(0.801,2.832)	1.339(0.650,2.760)
Q4	2.247(1.330,3.797) ***	2.169(1.189,3.956) *	1.997(0.937,4.255)

**Notes:** Model 1: Unadjusted; Model 2: Adjusted for age, body mass index (BMI); Model 3: Adjusted for age, body mass index (BMI), Education level, country of birth, HH Ref Person Gender, HH Ref Person Age, HH Ref Person Marital Status, age of menarche, number of pregnancies and whether at least one ovary was removed.

* p<0.05, p<0.01

*** p<0.001.

**Fig 2:**
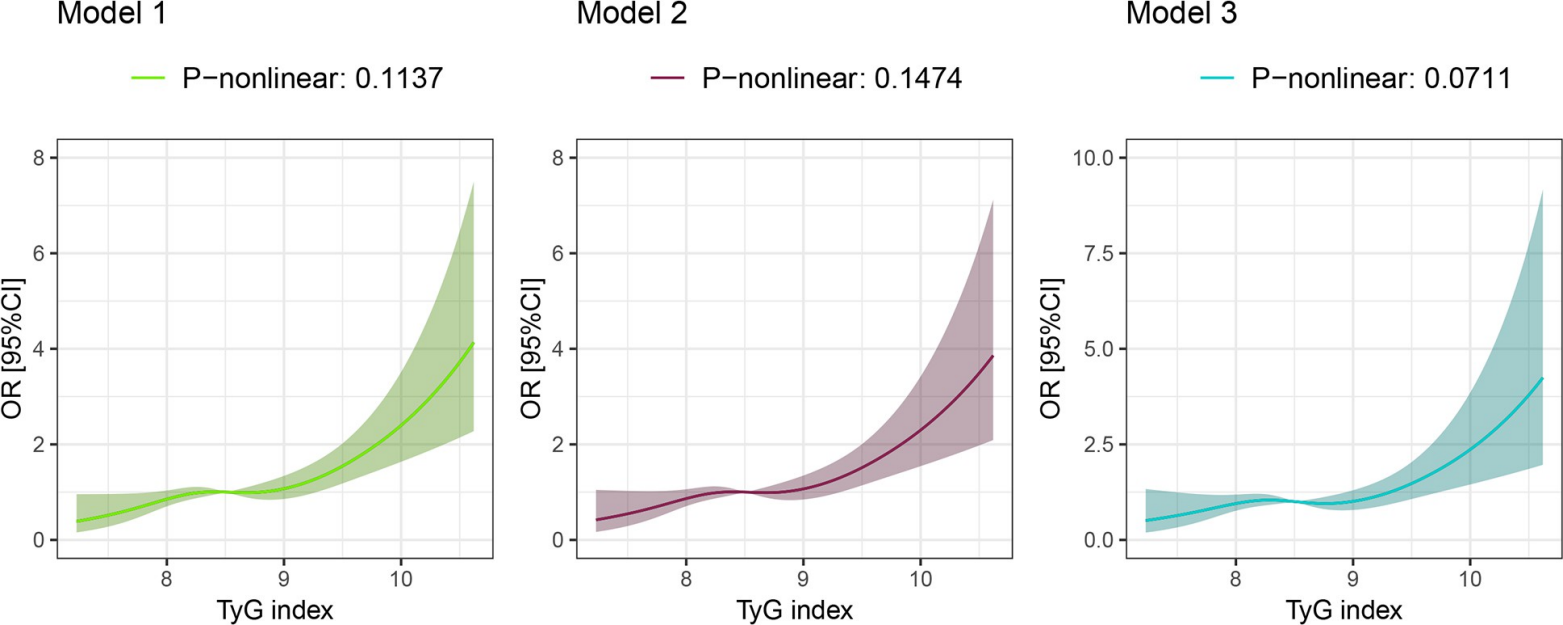
RCS analysis of the association between the TyG index and the risk of endometriosis.

As a continuous variable, the TyG index had a positive correlation with endometriosis. OR was 1.676 (95%: 1.293–2.172) in Model 1. 1.642 (95%: 1.224–2.204) in Model 2, and 1.52 (95%: 1.024–2.258) in Model 3, which was still significantly positive. Moreover, RCS analysis (Fig 2) revealed a non-linear correlation between the TyG index and endometriosis in the studied population (Model 1: P = 0.1137; Model 2: P = 0.1474; Model 3: P = 0.0711). The risk of endometriosis increased rapidly with the rise of the TyG index, especially the TyG index > 8.5.

**Fig 3:**
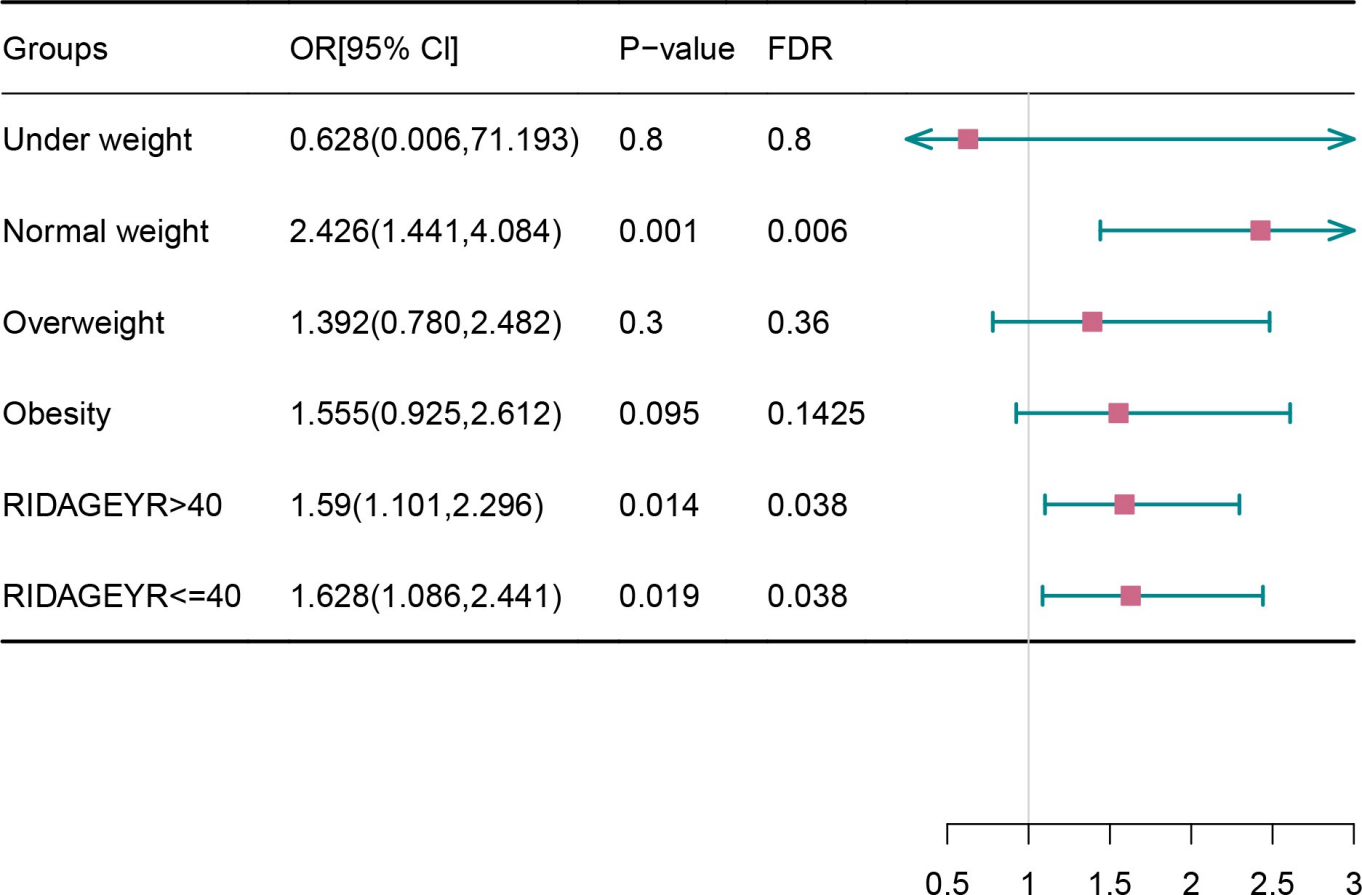
Subgroup analysis of the association between the TyG index and endometriosis.

As illustrated in Fig 3, the subgroup analysis elucidated that the TyG index retained a statistically significant association with the risk of endometriosis across subgroups stratified by age (≤40 years (OR [95% CI]: 1.628(1.086,2.441); P = 0.019) and >40 years (OR [95% CI]: 1.59(1.101,2.296); P = 0.014)) and BMI (OR [95% CI]: 2.426(1.441,4.084); P = 0.001). The TyG index remained significantly associated with endometriosis in all study populations.

IVW methods observed positive causal inference of IR to endometriosis (OR, 1.628; 95% ci, 0.061–0.913; p = .024) (Table 3). The sensitivity analysis revealed no heterogeneity (p for Cochran’s Q, 0.206) or pleiotropy (p for MR‐Egger intercept, 0.177) among these SNPs (Table 4).

**Table 3:** Mendelian randomization analysis between insulin resistance and endometriosis based on the ieu database.

method	SNPs	p-value	OR	Low	Upper
Inverse variance weighted	23	0.0248	1.628	0.061	0.913
MR Egger	23	0.5782	0.694	-1.631	0.901
Simple mode	23	0.2730	1.708	-0.398	1.469
Weighted median	23	0.1240	1.541	-0.118	0.984
Weighted mode	23	0.3122	1.521	-0.375	1.214

**Table 4:** Heterogeneity and pleiotropy of individual single nucleotide polymorphisms for Mendelian randomization.

		Heterogeneity		Pleiotropy	
Outcome	Exposure	IVW	P value	MR-Egger intercept	*P* value
EMS	IR	13.85	0.206	0.022	0.177

Abbreviations: IVW, inverse-variance weighted; EMS, endometriosis; IR, insulin resistance.

## 4.Discussion

TyG index has been identified as a reliable surrogate biomarker for IR [18]. Studies have demonstrated a link between the TyG index and the deterioration of cardiovascular disease, coronary artery disease, kidney stones, and chronic lung disease [19–22]. The pathogenesis of endometriosis is supported by diverse theoretical frameworks, such as the implantation theory, Selomic theory, and inflammatory diseases [23]. Nonetheless, the specific involvement of the TyG index in the pathogenesis of endometriosis has not been thoroughly investigated. Consequently, this study is to explore the relationship between TyG index and endometriosis using cross-sectional data from the NHANES database (1999–2006), with the goal of providing clinical evidence that deepens the understanding of endometriosis prevention and treatment strategies.

Among the models using multiple regression adjustment for covariates, we revealed a significant positive association of the TyG index with endometriosis. Notably, age, birthplace, whether an ovary was removed, and serum triglyceride levels were statistically significant variables in these analyses. Age is a key factor in metabolism, with an increasing risk of metabolic diseases including metabolic syndrome, IR, and diabetes observed as individuals grow older [24]. The aging process is characterized by persistent low inflammation, which is implicated in the pathophysiology of endometriosis [25]. Consequently, age significantly impacts the TyG index in the context of the pathogenesis and progression of endometriosis. The prevalence of endometriosis varies among individuals from different birthplaces. People from Mexico, Washington, and other regions in the United States are particularly at risk for developing endometriosis. Government departments in these areas must enhance efforts in disseminating information about endometriosis, thereby increasing public awareness and formulating comprehensive plans for prevention and treatment.

The RCS analysis also affirmed a robust and non-linear positive correlation between the TyG index and the incidence of endometriosis.

In the subgroup analysis by BMI, TyG index was significantly associated with endometriosis in populations with normal BMI and overweight, but not in obese patients. Normal and over-high BMI may have more pronounced impacts on the TyG index. Moreover, in the age-based subgroup analysis, age (≥20) was a statistically significant influencing factor. These results further reinforce the robustness of our conclusions, indicating a positive and non-linear correlation between the TyG index and endometriosis.

In addition, MR analysis showed a positive correlation between IR and endometriosis, and the heterogeneity and pleiotropy were P > .05 (Table 3). These results further suggest a causal link between IR and endometriosis. Our study demonstrated the link between IR and endometriosis both at the cross-sectional level and the genetic level. Therefore, the positive correlation between the TyG index and endometriosis was significant and stable.

The mechanism by which the TyG index affects the occurrence and development of endometriosis is not yet clearly defined. Prior research has indicated a positive correlation between the TyG index and arterial blood flow resistance index [26]. An increase in this resistance index may impede local blood circulation in the endometrium, causing the normal endometrium to flow back outside the uterine cavity, with hormonal fluctuations within the menstrual cycle. Moreover, the TyG index is a biologically reliable surrogate for IR. Obesity-mediated IR is strongly associated with the accumulation of pro-inflammatory macrophages and inflammatory responses [27]. Endometriosis is a chronic, hormone-dependent disease characterized by inflammation. Elevated TyG index causes the immune system to produce extensive immune-inflammatory factors, such as TNF-α and IL-1, which promote the development of endometriosis. Endometriosis caused by elevated TyG index may be associated with reduced local blood metabolism and neovascularization, which allow the ectopic endometrium to implant and grow repeatedly. Endometriosis is often associated with pain and infertility, and the high production of pro-inflammatory factors is associated with these two main symptoms of the disease. Some studies have shown that endometriosis is a manifestation of pelvic inflammatory disease, with abundant inflammatory factors in the pathologic examination of endometriosis, such as IL-6, macrophage migration inhibitory factor, TNF-α, IL-1b, IL-6, and IL-8 [28]. In addition, recent studies have highlighted that endometriosis may be related to biological mechanisms, such as implantation theory, Celomic theory, endometrial invagination, hormone receptors, and epigenetic regulators [23]. It is hypothesized that ectopic lesions resulting from retrograde menstruation of endometrial tissues may be influenced by immune dysfunction [29]. Therefore, we propose that the inflammatory environment associated with IR is a key risk factor for endometriosis. CA125 is the most recognized marker of endometriosis and there is still a lack of reliable biomarkers. TyG index, a biomarker of IR, may become a new reliable predictive marker of endometriosis. However, the specific biological mechanism needs to be validated by more studies. Consequently, further research is warranted to explore the mechanism of TyG index in the occurrence and development of endometriosis, to identify more targets for the prevention and treatment of endometriosis. Further research on the target of the TyG index may provide more theoretical basis and data support for clinical research on targeted drug therapies for endometriosis.

### 4.1 Limitations

Our findings suggest a positive association between TyG index and endometriosis. However, our study was confined to participants in only four cycles, indicating the necessity for. a larger cohort. Second, the cross-sectional nature of this study, based on observational survey data, restricted the findings to an association rather than a causal link. Moreover, more than half of the subjects were excluded due to incomplete data, which may introduce bias into the results. Given these limitations, further studies are warranted to delve into the intricate molecular mechanisms by which the TyG index affects the onset and progression of endometriosis.

## 5.Conclusion

TyG index has a robust positive correlation with endometriosis. With the increase of the TyG index, the risk of endometriosis also increases significantly. Age and birthplace are significant factors influencing the relationship.

## Abbreviations

NHANES: Nutrition Examination Survey
TyG: The triglyceride glucose index
RCS: Restricted cubic spline models
TyG index: Triglyceride-glucose index
RHQ: Reproductive health questionnaire
SD: Standard deviation
IQR: Interquartile range
OR: odds ratio
MR: Mendelian randomization
